# Socioeconomic and demographic factors associated with caesarean section delivery in Southern Ghana: evidence from INDEPTH Network member site

**DOI:** 10.1186/s12884-018-2039-z

**Published:** 2018-10-16

**Authors:** Alfred Kwesi Manyeh, Alberta Amu, David Etsey Akpakli, John Williams, Margarete Gyapong

**Affiliations:** 1 Dodowa Health Research Centre, P. O. Box. DD1, Dodowa, Accra Ghana; 2 Division of Epidemiology and Biostatistics, School of Public Health, University of the Witwatersrand, Parktown, Johannesburg, South Africa; 3 Ghana Health Service, Accra, Ghana; 4 Centre for Health Policy and Implementation Research, Institute for Health Research, University of Health and Allied Sciences, Volta Region, Ho, Ghana

**Keywords:** Caesarean section, INDEPTH network, Dodowa, Ghana

## Abstract

**Background:**

In recent years, caesarean section rates continue to evoke worldwide concern because of their steady increase, lack of consensus on the appropriate caesarean section rate and the associated short- and long-term risks.

This study sought to identify the rate of caesarean section and associated factors in two districts in rural southern Ghana.

**Methods:**

Pregnancy, birth, and socio-demographic information of 4948 women who gave birth between 2011 and 2013 were obtained from the database of Dodowa Health and Demographic Surveillance System. The rate of C-section was determined and the associations between independent and dependent variables were explored using logistic regression. The analyses were done in STATA 14.2 at 95% confidence interval.

**Results:**

The overall C-section rate for the study period was 6.59%. Women aged 30–34 years were more than twice likely to have C-section compared to those < 20 year (OR: 2.16, 95% CI: 1.20–3.90). However, women aged 34 years and above were more than thrice likely to undergo C-section compared to those < 20 year (OR: 3.73, 95% CI: 1.45–5.17).

The odds of having C-section was 65 and 79% higher for participants with Primary and Junior High level schooling respectively (OR: 1.65, 95% CI: 1.08–2.51, OR:1.79, 95%CI: 1.19–2.70). The likelihood of having C-section delivery reduced by 60, 37, and 35% for women with parities 2, 3 and 3+ respectively (OR:0.60, 95% CI: 0.43–0.83, OR: 0.37, 95% CI: 0.25–0.56, OR:0.35, 95% CI: 0.25–0.54). There were increased odds of 36, 52, 83% for women who belong to poorer, middle, and richer wealth quintiles respectively (OR: 1.36, 95%CI: 0.85–2.18, OR: 1.52, 95% CI: 0.97–2.37, OR: 1.83, 95% CI: 1.20–2.80). Participants who belonged to the richest wealth quintile were more than 2 times more likely to have C-section delivery (OR: 2.14, 95%CI: 1.43–3.20). The odds of having C-section delivery reduced by 76% for women from Ningo-Prampram district (OR: 0.76, 95% CI: 0.59.0.96). Women whose household heads have Junior High level and above of education were 45% more likely to have C-section delivery (OR: 1.45, 95% CI: 1.09–1.93).

**Conclusion:**

Age of mother, educational level, parity, household socioeconomic status, district of residence, and level of education of household head are associated with caesarean section delivery.

## Background

Accessibility of comprehensive emergency obstetric care (including caesarean section) is crucial to averting the estimated 2.9 million neonatal and 287,000 maternal mortalities that occur worldwide every year [1, 2].

Caesarean section (C-section), is one of the oldest and regularly used surgical procedures in Obstetrics by which fetus is delivered through an abdominal and uterine incision [3–5]. It has been shown that, when C-section is appropriately used, it can improve both infant and maternal health outcomes [6, 7]. According to the World Health Organization (WHO), C-section is a vital treatment in pregnancy [8]. However, the potential risk of C-section may outweigh the benefits when it is used inappropriately [6, 7].

In recent years, the number of C-section deliveries has been increasing in developed and developing countries [9–11]. This increase has however not been clinically justified. This worldwide increase in C-section has become a major public health issue due to potential maternal and perinatal risks, inequality of access and cost involved [12–17]. According to the WHO guidelines, no region is justified for having the rate of C-section more than 10–15% [6, 18]. Despite this WHO guidelines, studies show that the rates of C-sections are high in developing countries [17, 19, 20]. A WHO reports shows that, the global average of C-section rate between 1990 and 2014 increased from 12.4 to 18.6% [21]. While the C-sections rate varies between 12 and 86% across studies done in developed countries [22–25] that of developing countries ranges between 2 and 39% [8, 22, 26–29]. This again shows the cause for concern and hence the need to explore the reasons for the increasing rates in C-section delivery [6, 18].

Although there is no evidence of benefits of C-section to mothers or babies who do not need it [30], however, C-section like any surgery, has complications which may persist for a long period after the current delivery and affect the health of the woman, her child, and future pregnancies. According to literature, the global increase in C-section is associated with uterine rupture such that, women with prior who undergo the procedure are more likely to have uterine rupture in future pregnancies [31–35]. The risk of uterine rupture due to prior C-section are higher in population with limited access to comprehensive obstetric care [17, 36, 37].

Studies have attribute the increase in C-sections to multiple factors ranging from the type of health facility, socio-demographic characteristics to maternal health of women [38–43]. These factors include maternal age [38–40], birth order [44], birth weight [45], place of residence [14], socioeconomic status, maternal educational level [38, 46–48], former C-section [41–43], obstetric complications [49], maternal request [50], and income level [38, 48]. These factors also vary among different populations [16].

The global increase in the rate of the provision of C-section is reflected in Ghana. According to the 2014 Ghana Demographic and Health Survey (GDHS) survey, the C-section rate in Ghana increased from 4.5 to 6.4% between 1990 and 2005 [51]. In 2014, the GDHS reported that 13% of births are delivered by C-section, an increase from 7% in 2008 [52]. Delivery by C-section is highest among births to women aged 35–49 (17%); first-parity births (18%); births for whom women had more than three Antenatal Care (ANC) attendance (15%); deliveries in urban areas (19%) and in the Greater Accra region (23%); births to women with secondary school level and above education (27%), and those with richest socioeconomic status (28%) [52].

Primary C-section usually determines the future obstetric course of any woman [53]. Hence there is a need to assess the rate of C-sections and factors contributing to C-section delivery in the context of Ghana using population based data. The existence of a Health and Demographic Surveillance System provided a unique opportunity to study the factors contributing to C-section delivery at the population level. This is because most studies on C-section used hospital based data which were subject to selection bias. The study of determinants of C-section in a population makes it possible to explore how different elements contribute to the decision to perform a C-section. Therefore, this study aimed to examine the rate of C-section delivery and to explore factors associated with the procedure in two rural districts in Ghana.

## Methods

### Study area

This study used secondary data from the Dodowa Health and Demographic Surveillance System (DHDSS). The DHDSS site is in the Shai-Osudoku and Ningo-Prampram districts of the Greater Accra Region of Ghana. The two districts together have a total population of 115, 754 people in 380 communities living in 23, 647 households. A detailed description of the DHDSS and its operations can be found elsewhere [54–56]. Health care service delivery in the DHDSS is provided from government hospitals, health centres, clinics, community-based health and planning services (CHPS) compounds/zones, and non-governmental health facilities [54].

### Study population

All mothers who were resident in study area and had a delivery between January 1, 2011 to December 31, 2013 were included in the study. Therefore, all mothers who were outside the study area and those who delivered outside the study date were excluded. A total of 4, 948 women were included in the study.

### Variables

#### Dependent variable

The dependent variable is type of delivery and it was dichotomous (coded as 1 if the respondents underwent C-section delivery and 0 if otherwise).

#### Independent variables

From available data, the exposure variables included are maternal age, educational level, marital status, parity, timing of ANC visit, place of delivery, child gender and weight at birth, educational level of household head, district of residence and socio-economic status.

The socio-economic status is calculated using the household’s social status, ownership of assets, availability of utilities among others using weights derived through a principal component analysis (PCA) [57]. The socio-economic status is a proxy measure of a household’s long term standard of living [54]. The proxies from the PCA were divided into five quintiles; poorest, poorer, middle, richer and richest.

### Statistical methods

A descriptive analysis of socio-demographic characteristics of the participants was carried out. The associations between the exposure variables and the outcome of interest were explored in a crude and adjusted ordered logistics regression model. The exposure variables that were significant at *p* <  0.05 in the crude model were entered together into an adjusted model. Stata version 14.2 was used for the analysis and the findings were presented in tables with summary statistics at 95% confidence intervals (CI).

## Results

### Socio-demographic characteristics

Table 1 presents the socio-demographic characteristics of 4948 study participants. The mean age of the study participants was 28 years (SD = 7.06).

**Table 1:** Socio-Demographic Characteristics of the study participants

Age group	Frequency^a^	Percentage
< 20	552	11.16
20–24	1151	23.26
25–29	1288	26.03
30–34	1061	21.44
34 +	896	18.11
Mean = 28.01 (SD = 7.06)		
Occupation		
Unemployed	1125	22.74
Farmer	855	17.28
Artisan	608	12.29
Trader	1508	30.48
Civil Servant	84	1.7
Student	669	13.52
Others	99	2
Level of education		
No Education	1333	26.94
Primary	1502	30.36
JHS /Middle school	1691	34.18
SHS and above	422	8.53
Marital status		
Single	1325	27.2
Married	1010	20.73
Separated/Divorced	99	2.03
Cohabiting	2437	50.03
Parity		
Parity 1	1315	26.58
Parity 2	1183	23.91
Parity 3	940	19
Parity 3+	1510	30.52
Timing of ANC visit		
First trimester	2732	55.21
Second trimester	1980	40.02
Third trimester	236	4.77
Type of delivery		
Normal delivery	4621	93.41
C-Section delivery	326	6.59
Child weight at birth		
Low birth weight	232	4.69
Normal birth weight	2760	55.78
Not Weighed	1956	39.53
Delivery place		
Health Facility	3359	67.98
Outside Health Facility	1582	32.02
Child gender		
Female	2332	47.13
Male	2616	52.87
Household heads education		
No education/Primary	2614	52.83
JHS and above	2334	47.17
Household heads gender		
Female	1893	38.26
Male	3055	61.74
District of residence		
Shai-Osudoku	2165	43.76
Ningo-Prampram	2783	56.24

*n* = 4948; *SD* Standard Deviation; ^a^due to missing data, the number of participants is may not be 4948 in all categories

While teenagers (< 20 years) contributed the least proportion of the study participants (11.16%), the 25–29 age group formed the highest proportion (26.03%) followed by the 20–24 and the 30–34 years’ groups which accounted for 23.26 and 21.44% respectively.

While 30.48% of the study participants were petty traders, 22.74 and 17.28% were unemployed and farmers respectively. Students formed 13.52% of the study participants. Mothers with Junior high school and primary school level contributed 34.18 and 30.36% respectively.

Participants without formal education accounted for 26.94% of the study’s participants. A large proportion of the study participants had their marital status as cohabiting (50.03%) while those married, single, and separated /widowed were 20.73, 27.20, and 2.03% respectively.

Participants with parity 3 or more formed 30.52% of participants while those with parity 1 and 2 were 26.56, and 23.91% respectively.

More than half (55.21%) of the study respondents started ANC visit in the first trimester of gestation. Respondents who initiated ANC attendance in the second and third trimesters formed 40.02 and 4.77% of the sample respectively.

The overall C-section delivery rate for the study period was 6.59%. The C-section rate for Shai-Osudoku District was 7.81% and that of Ningo-Prampram was 5.64%%.

The result of this study shows that 55.78% of the babies were of normal weight. Majority (67.98%) of the study participants delivered in a health facility.

Greater proportion of the babies born (52.87%) were males. More than half (52.83%) of the household heads have no education or primary level of education. As much as 56.24% of the participants were from the Ningo-Prampram district while 43.76% were from Shai-Osudoku district.

### Crude and adjusted odds ratio of determinants of C-section delivery

Table 2 presents the crude and adjusted Odds Ratio (OR) at 95% Confidence Interval (CI) of socioeconomic and demographic factors associated with C-section delivery in Dodowa Health and Demographic Surveillance site.

**Table 2:** Crude and adjusted odd ratios of determinates of C-section delivery

Variable	Crude		Adjusted^b^	
	Odd Ratio (95% CI)	*P*-value	Odd Ratio (95% CI)	*P*-value
Age group				
< 20	1.00		1.00	
20–24	1.06 (0.66–1.70)	0.825	1.05 (0.64–1.74)	0.839
25–29	1.43 (0.91–2.24)	0.121	1.42 (0.83–2.43)	0.202
30–34	1.81 (1.15–2.84)^a^	0.010	2.16 (1.20–3.90)^a^	0.010
34 +	1.74 (1.10–2.77)^a^	0.019	3.73 (1.45–5.17)^a^	0.002
Level of Education				
No Education	1.00		1.00	
Primary	1.75 (1.18–2.59)^a^	0.006	1.65 (1.08–2.51)^a^	0.019
Junior High school	2.79 (1.93–4.01)^a^	< 0.001	1.79 (1.19–2.70)^a^	0.005
Senior High School and above	7.88 (5.28–11.76)^a^	< 0.001	3.53 (2.17–5.73)^a^	< 0.001
Occupation				
Unemployed	1.00		1.00	
Farmer	1.08 (0.72–1.62)	0.706	1.01 (0.71–1.71)	0.678
Artisan	2.37 (1.63–3.44)^a^	< 0.001	1.48 (0.99–2.20)	0.055
Trader	1.28 (0.91–1.80)	0.159	1.36 (0.95–1.95)	0.095
Civil Servant	3.56 (1.86–6.83)^a^	< 0.001	0.78 (0.38–1.59)	0.496
Student	1.34 (0.89–2.02)	0.159	1.26 (0.77–2.05)	0.363
Others	2.43 (1.23–4.81)^a^	0.011	2.31 (1.10–4.85)^a^	0.026
Marital Status				
Single	1.00		1.00	
Married	1.64 (1.21–2.22)^a^	0.001	1.26 (0.851–1.85)	0.247
Separated/Divorced	0.96 (0.41–2.27)	0.932	1.00 (0.37–2.27)	0.856
Cohabiting	0.84 (0.63–1.11)	0.224	0.94 (0.66–1.32)	0.705
Parity				
Parity 1	1.00		1.00	
Parity 2	0.74 (0.55–0.99)^a^	0.045	0.60 (0.43–0.83)^a^	0.002
Parity 3	0.57 (0.41–0.80)^a^	0.001	0.37 (0.25–0.56)^a^	< 0.001
Parity 3+	0.51 (0.37–0.68)^a^	< 0.001	0.35 (0.23–0.54)^a^	< 0.001
Timing of ANC visit				
First trimester	1.00		1.00	
Second trimester	0.67 (0.52–0.85)^a^	< 0.001	0.81 (0.63–1.05)	0.106
Third trimester	0.15 (0.05–0.47)^a^	< 0.001	0.24 (0.07–0.76)^a^	0.015
Socio Economic Status				
Poorest	1.00		1.00	
Poorer	1.12 (0.71–1.77)	0.627	1.36 (0.85–2.18)	0.205
Middle	1.44 (0.93–2.23)	0.099	1.52 (0.97–2.37)	0.069
Richer	2.15 (1.43–3.23)^a^	< 0.001	1.83 (1.20–2.80)^a^	0.005
Richest	3.84 (2.62–5.63)^a^	< 0.001	2.14 (1.43–3.20)^a^	< 0.001
District of residence				
Shai-Osudoku	1.00		1.00	
Ningo-Prampram	0.71 (0.56–0.88)^a^	0.002	0.76 (0.59–0.96)^a^	0.024
Household heads education				
No education/Primary	1.00		1.00	
Junior High School and above	2.65 (2.08–3.38)^a^	< 0.001	1.45 (1.09–1.93)^a^	0.010
Household heads gender				
Female	1.00		1.00	
Male	1.29 (1.01–1.64)	0.038		
Child weight at birth				
Low birth weight	1.00			
Normal birth weight	0.80 (0.48–1.34)	0.404		
Child Gender				
Female	1.00			
Male	1.12 (0.89–1.41)	0.32		

*SD* standard deviation, ^a^statistical significant, ^b^Correct classification rate = 93.41%

In the crude model, there was a statistically significant association between maternal age and C-section delivery.

The odds of having C-section delivery by women aged 20–24 and 25–29 years is 6 and 43% respectively more likely compared to those aged < 20 years (OR: 1.06, 95% CI: 0.66–1.70, OR: 1.43, 95% CI: 0.91–2.24). Women aged 30–34 and 34+ years were 81 and 74% respectively more likely to have C-section delivery compared to those aged < 20 years (OR; 1.80, 95% CI: 1.15–2.84, OR:1.74, 95% CI: 1.10–2.77). This is statistically significant.

A similar relationship is observed after adjusting other explanatory variables such that, the odds of having C-section went up with increasing maternal age. Women aged 30–34 and 34+ years were more than twice and thrice more likely respectively to have C-section compared to those aged < 20 years (OR:2.16, 95% CI: 1.20–3.90, OR: 3.73, 95% CI: 1.45–5.17). This was statistically significant.

The results further revealed that, the odds of women having C-section delivery went up with increasing level of education in both the crude and adjusted models. In the crude model, odds of women with primary level of formal education having C-section delivery was 75% higher compared to those with no education (OR:1.75, 95% CI: 1.18–2.59). The odds of women with Junior High School (JHS) level of education having C-section delivery as compared to those with no education is almost three times more likely (OR: 2.79, 95% CI: 1.93–4.01). Again, the odds of participants with Senior High level of schooling having C-section delivery was eight times more likely compared to those with no formal education (OR: 7.88, 95% CI: 5.28–11.76).

Holding other variables constant, the odds of having C-section was 65 and 79% higher for participants with Primary and JHS level of schooling respectively compared to those with no education (OR: 1.65, 95%CI: 1.08–2.51, OR:1.79, 95% CI: 1.19–2.70).

In the crude logistics regression model, maternal occupation was statistically significantly associated with C-section. Women who are artisans were more than twice more likely to have C-section compared to those unemployed (OR: 2.37, 95% CI: 1.63–3.44). This was also statistically significant. Women who were civil servants and women who were engaged in other forms of occupation were more than three and two times more likely to undergo C-sections compared with those who were unemployed (OR: 3.56, 95% CI: 1.86–6.83, OR:2.43, 95% CI:1.23–4.81). This is also statistically significant.

Women who were married were 64% more likely to have C-section delivery (OR: 1.64, 95% CI: 1.21–2.22) compared to those who are single. This again was statistically significant. For participants with marital status as separated/divorced and cohabiting, the odds of having a C-section reduced to 96 and 84% respectively compared to those who were single (OR: 0.96, 95% CI: 0.41–2.27, OR:0.84, 95% CI: 0.63–1.11). In the adjusted model, participants who were married had increased odds of 26% of having C-section delivery compared to those who were single (OR: 1.26, 95% CI: 0.85–1.85).

In the crude model, study participants who started ANC visit in the second and third trimesters were 67 and 15% respectively less likely to have a C-section compared with those who started their ANC visit in the first trimester (OR: 0.67, 95% CI: 0.52–0.85, OR: 0.15, 95% CI: 0.05–0.68). After adjusting other explanatory variables, women who started ANC visit in the third trimester were 24% less likely to have C-section compared to those who initiated their ANC visit in the first trimester of pregnancy. This was statistically significant (OR: 0.24, 95% CI: 0.07–0.76).

The odds of having C-section delivery reduced significantly with increasing parity. There was reduced odds of 74, 57 and 51% of women with parities 2, 3 and 3+ respectively having C-section delivery compared to those with parity 1 (OR: 0.74, 95% CI: 0.55–0.99, OR: 0.57, 95% CI: 0.41–0.80, OR:0.51, 95% CI: 0.37–0.68). In the adjusted model, a similar relationship was observed such that the odds of having C-section delivery reduced with increasing parity. Thus, there was reduced odds of 60, 37, and 35% for women with parities 2, 3 and 3+ respectively compared with those with parity 1(OR:0.60, 95% CI: 0.43–0.83, OR: 0.37, 95% CI: 0.25–0.56, OR:0.35, 95% CI: 0.25–0.54).

The odds of having C-section went up with increasing socioeconomic status. In the crude analysis, participants with middle wealth quintile were 44% more likely to have C-section (OR: 1.44, 95%CI:0.93–2.23) compared to those in the poorest group. Participants who belong to the richer and richest quintiles were more than two times and three times more likely to have C-section delivery compared to those who belong to the poorest group (OR: 2.15, 95% CI: 1.43–3.23, OR: 3.84, 955 CI: 2.62–5.63). Participants’ socioeconomic status continued to be increasingly significantly associated with C-section delivery after adjusting other confounding variables in the adjusted model. There were increased odds of 36, 52, 83% for women who belong to poorer, middle, and richer wealth quintiles respectively (OR: 1.36, 95% CI: 0.85–2.18, OR: 1.52, 95% CI: 0.97–2.37, OR: 1.83, 95% CI: 1.20–2.80). Participants who belong to the richest wealth quintile were more than two times more likely to have C-section delivery compared to those who were in the poorest category (OR: 2.14, 95% CI: 1.43–3.20).

The district where participant resides was significantly associated with C-section delivery such that, there was reduced odds of 71 and 76% in the crude and adjusted models respectively for women from Ningo-Prampram district compared to those from Shai-Osudoku district (OR:0.71, 95% CI: 0.56–0.88, OR:0.76, 95% CI: 0.59.0.96).

While there were increased odds of participants giving birth to male babies having C-section delivery (OR: 1.12, 95%CI: 0.89–1.41) compared to those with female babies, there was reduced odds of 80% of having C-section for participants who gave birth to normal weight babies in the crude model (OR: 0.80, 95% CI: 0.48–1.34) compared to those with low birth weight.

There was a statistically significant association between the level of education of household head and C-section such that participants whose household heads have JHS or more education were more than two times more likely to have C-section in the crude model (OR: 2.65, 95% CI: 2.08–3.38) compared to those whose heads had primary/no formal education. In the adjusted model, women whose household heads had JHS and above level of formal education were 45% more likely to have C-section delivery compared to those with primary / no formal education (OR: 1.45, 95% CI: 1.09–1.93).

## Discussion

Despite the relevance and worldwide interest in this topic, this is the first study in Ghana to have used population based data at the district level to identify the rate and explore factors associated with C-section delivery. Our analysis of data from 4948 research participants revealed that C-section delivery is associated with maternal age, level of education, occupation, parity and ANC visit. The study also showed that other variables associated with C-section delivery include socioeconomic status, district of residence and level of education of household head.

The overall C-section rate of 7% found by the current study is lower than the national rate of 13% reported in 2014 by GHDS [52]. The current C-section rate is also lower than the WHO recommended rate of 10–15% [6, 18]. It is however similar to the 7.3% reported by Betrán AP et al. for Africa [58] and higher than the 3% estimated for Western Africa [58].

The findings of our study is consistent with the results of previous studies such that factors such as level of education of women, socio-economic status [38, 46–48, 59, 60], maternal occupation status [33], maternal age [38–40, 52, 59, 61], parity [44, 59], place of residence [14], and level of education of household head [60] are associated with C-section delivery.

The findings of our study also confirmed the results of GHDS 2014 which suggested that C-section delivery is associated with advanced maternal age (35–49 years), order of births (parity), ANC visit, maternal level of education, and socioeconomic status [52]. The relationship between advanced maternal age and some adverse pregnancy outcomes and higher risk of medical conditions like hypertension and diabetes as shown by other studies [60, 62] could explain why increasing maternal age was associated with the increased odds of having C-section delivery in our study.

The lower likelihood of C-section delivery among study participants with increasing parity could mean that many women with three caesarean sections do not get pregnant again to avoid further C-section delivery as established by Nilsen C et al. [63]. This could also be due to the fact that once the woman’s pelvis has been tested with a previous pregnancy and delivery, subsequent deliveries tend to be less risky until she reaches her fifth delivery (grand-multipara) when the risk increases again as shown in a previous study [64].

The likelihood of mothers in Shai-Osudoku district having C-section delivery as compared to those from Ningo-Prampram district could be explained by the availability of a district hospital in Shai- Osudoku and three other referral hospital in districts that share boundary with Shai-Osudoku district, therefore providing more ready access to C-sections. This finding corroborates results of other studies which suggest that C-section delivery is associated with availability of and access to a medical facility [65–67].

### Strengths and limitations

The strengths of this study were its data quality and large sample size. Also, being a community based study with focus on rural communities which are priority for public health interventions was a strength of the study. This notwithstanding, the study had a few limitations. The secondary data used did not include other important variables on maternal health status, evidence of whether the earlier delivery was by caesarean and fetal characteristics that may influence the risk of C-section. The data used was also not a nationally representative one. This is because the two districts cannot be true representative of 216 districts in Ghana hence the limit in the generalizability of the findings.

## Conclusion

The study established that the overall C-section rate in DHDSS site is 6.59%. The findings reinforce the evidence that the odds of having C-section delivery increases with advancing maternal age, level of education and household socioeconomic status. Parity, district of residence, and level of education of household head are other variables that are associated with C-section delivery. To understand other factors influencing C-section delivery and to design an appropriate intervention, we recommend further qualitative research in this area.

## Abbreviations

ANC: Antenatal Care
CHPS: Community-based Health Planning and Services
CI: Confidence Intervals
DHDSS: Dodowa Health and Demographic Surveillance System
DHRC: Dodowa Health Research Centre
EOC: Emergency Obstetric Care
GDHS: Ghana Demographic Health Survey
JHS: Junior High school
MDG: Millennium Development Goal
OR: Odd Ratio
PCA: Principal component analysis
