# Correction to: Socioeconomic and demographic factors associated with caesarean section delivery in Southern Ghana: evidence from INDEPTH Network member site

**DOI:** 10.1186/s12884-018-2118-1

**Published:** 2019-01-08

**Authors:** Alfred Kwesi Manyeh, Alberta Amu, David Etsey Akpakli, John Williams, Margaret Gyapong

**Affiliations:** 1 Dodowa Health Research Centre, P. O. Box. DD1, Dodowa, Accra, Ghana; 2 Division of Epidemiology and Biostatistics, School of Public Health, University of the Witwatersrand, Parktown, Johannesburg, South Africa; 3 Ghana Health Service, Accra, Ghana; 4 Centre for Health Policy and Implementation Research, Institute for Health Research, University of Health and Allied Sciences, Volta Region Ho, Ghana

**Correction to: BMC Pregnancy and Childbirth (2018) 18:405**

**https://doi.org/10.1186/s12884-018-2039-z**

Following publication of the original article [1], the author reported the following errors:

1. The correct name of the last author is Margaret Gyapong and not Margarete Gyapong.

2. The word “ordered” in the 3rd line under statistical methods should be removed.

The original article has been corrected.
